# Moderate Risk of Hepatitis B Virus Reactivation in HBsAg^−^/HBcAb^+^ Carriers Receiving Rituximab for Rheumatoid Arthritis

**DOI:** 10.1038/s41598-020-59406-4

**Published:** 2020-02-12

**Authors:** Meng Hsuan Kuo, Chih-Wei Tseng, Chi-Hui Lee, Chien-Hsueh Tung, Kuo-Chih Tseng, Ning-Sheng Lai

**Affiliations:** 1 Department of Pharmacy, Dalin Tzu Chi Hospital, Buddhist Tzu Chi Medical Foundation, Chia-Yi, Taiwan; 2 School of Medicine, Tzuchi University, Hualien, Taiwan; 3 Division of Gastroenterology, Department of Internal Medicine, Dalin Tzu Chi Hospital, Buddhist Tzu Chi Medical Foundation, Chia-Yi, Taiwan; 4 Division of Rheumatology, Department of Internal Medicine, Dalin Tzu Chi Hospital, Buddhist Tzu Chi Medical Foundation, Chia-Yi, Taiwan

**Keywords:** Hepatitis B, Rheumatoid arthritis

## Abstract

To investigate the incidence and risk factors of hepatitis B virus (HBV) reactivation in HBV surface antigen (HBsAg)^−^/ HBV core antibody (HBcAb)^+^ patients who underwent rituximab (RTX) therapy for rheumatoid arthritis (RA). From January 2000 through December 2017, a total of 134 RA patients with various HBV serostatuses who received RTX at Dalin Tzu Chi Hospital were screened. Finally, 50 HBsAg^−^/HBcAb^+^ patients were enrolled in this retrospective study. Baseline characteristics, comedications, and the occurrence of HBV reactivation were recorded. Four HBsAg^−^/HBcAb^+^ RA patients (8%; 4/50) experienced HBV reactivation after treatment with RTX. Hepatitis flare-up occurred in 2 of these 4 patients, with a fatal outcome in one. HBV reactivation occurred approximately 1–4 years after the first dose of RTX and 0.5–1.5 years after the last one. In HBsAg^−^/HBcAb^+^ patients, HBV reactivation was significantly more common in those who were HBV surface antibody (HBsAb)^−^ at baseline than in those who were HBsAb^+^ (30% vs 4%; *p* = 0.02). A history of adalimumab use was associated with HBV reactivation (100% vs 39%; *p* = 0.02). A moderate risk of HBV reactivation was observed in HBsAg^−^/HBcAb^+^ RA patients receiving RTX therapy. The reactivation may induce acute hepatitis and even death. To reduce the risk of HBV reactivation, regular monitoring of liver function is insufficient; monitoring of viral load and HBsAg or prophylaxis with antiviral therapy should be considered.

## Introduction

Rheumatoid arthritis (RA) is a chronic systemic inflammatory autoimmune disease characterized by proliferative synovitis. RA treatment aims to control both inflammation and pain and to reduce disability related to the proper use of disease-modifying antirheumatic drugs (DMARDs), categorized as synthetic DMARDs (sDMARDs) and biological DMARDs (bDMARDs)^1^. The use of bDMARDs targeting key components of the immune system can effectively inhibit the pathologic inflammation cascade that causes RA symptoms and subsequent joint destruction^2^. However, treatment with bDMARDs makes patients more susceptible to bacterial infections and potential reactivation of viral infections, such as hepatitis B or C, herpes, and varicella zoster^3–5^.

HBV reactivation is an emerging problem and a potentially life-threatening complication in patients receiving chemotherapy or immunosuppressive therapy, especially in epidemic regions. Among those bDMARDs currently used, rituximab (RTX) has received particular attention. This chimeric murine/human monoclonal antibody targets CD20-positive cells, causing long-lasting B lymphocyte depletion. As a result, rituximab administration is associated with an increased risk of HBV reactivation in patients with chronic infection with hepatitis B surface antigen (HBsAg)-positive HBV or hepatitis B core antibody (HBcAb)-positive HBV^6^. Therefore, the current RTX label contains a black-box warning about the risk of HBV reactivation, recommending that all patients be screened for HBV serum markers before starting RTX therapy and be monitored during and after treatment.

A higher incidence of HBV reactivation in lymphoma has been reported in HBsAg^−^/HBcAb^+^ patients receiving RTX-containing chemotherapy (24–45%)^7–9^ than in those receiving conventional chemotherapy (1–3%)^10^. The guidelines recommended anti-HBV prophylaxis for HBsAg−/HBcAb+ patients receiving anti-CD20 antibody therapy (e.g., rituximab in the oncohematological setting)^11,12^. However, those suggestions were based on the evidence from rituximab studies in the oncohematological setting (risk > 10%)^12^. The use of RTX differs between patients with RA and lymphoma in terms of dosage, regimen, and concomitant medications. Reports of HBV reactivation during treatment for rheumatic diseases are inconsistent. Two studies including a total of 26 HBsAg^−^/HBcAb^+^ RA patients reported no HBV reactivation after RTX^13,14^. In a cohort study conducted in a regional hospital in Taiwan, approximately 9.1% of HBsAg^−^/HBcAb^+^ RA patients (4/44) experienced HBV reactivation approximately 2 years after receiving RTX^15^. In a retrospective multicenter Italian study, the HBV reactivation rate was low (3%; 1/33) in HBsAg^−^/HBcAb^+^ patients^16^. Only one patient became positive for HBV DNA after 6 months of RTX treatment and was effectively treated with lamivudine, which prevented hepatitis flare-ups.

Because data regarding HBV reactivation in HBsAg^−^/HBcAb^+^ RA patients treated with RTX are limited, the optimal management protocol is still unclear. An understanding of the risk of HBV reactivation in patients is necessary for physicians to decide whether to administer antiviral therapy and whether monitoring HBV status is necessary. Here we aimed to determine the incidence of HBV reactivation and hepatitis flare-ups in HBsAg^−^/HBcAb^+^ patients after receiving RTX therapy for RA at our hospital.

## Results

### Demographics and baseline clinical features of RA patients with HBsAg^−^/HBcAb^+^

**Figure 1:**
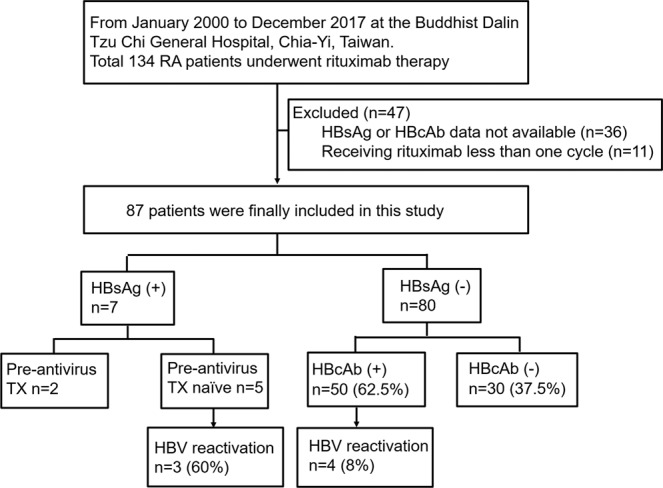
Flow diagram of study cohort characteristics. Flowchart shows the hepatitis B virus (HBV) infection serostatus distribution at baseline and the occurrence of HBV reactivation in rheumatoid arthritis (RA) patients treated with rituximab. HBsAg, HBV surface antigen; HBcAb, HBV core antibody; HBsAb, HBV surface antibody; NA, not available; TX, treatment.

Table 1 shows the clinical, serological, virologic characteristics and therapeutic regimens of patients with HBsAg^−^/HBcAb^+^. A total of 50 HBsAg^−^/HBcAb^+^ RA patients who received RTX therapy were enrolled in this study. Among these patients, the HBV reactivation rate was 8% (Fig. 1). The average age was 69.1 ± 11.0 years, and 37 of the patients (74%) were female (Table 1).

**Table 1:** Patient baseline characteristics.

Determinants^#^	HBsAg^−^/HBcAb^+^	HBV Reactivation^+^	HBV Reactivation^−^	*p*^†^
	N = 50	N = 4	N = 46	
Age (years)	69.1 ± 11	77.5 ± 9.7	68.4 ± 11.3	0.12
Female	37 (74%)	4 (100%)	33 (72%)	0.56
HBsAb				
Positive	26 (52%)	1 (25%)	25 (54%)	0.02
Negative	10 (20%)	3 (75%)	7 (15%)	
NA	14 (28%)	0 (0%)	14 (30%)	
Anti-HCV				
Positive	9 (18%)	1 (25%)	8 (17%)	0.56
Negative	41 (82%)	3 (75%)	38 (83%)	
RTX cycle	5.7 ± 4.1	3.5 ± 1.7	5.8 ± 4.2	0.28
Disease follow-up (years)	3.9 ± 2.5	3 ± 2.5	4 ± 2.6	0.47
BMI	24.1 ± 5.1	21.4 ± 7.7	24.4 ± 4.9	0.12
Hypertension	14 (28%)	1 (25%)	13 (28%)	1
Hyperlipidemia	7 (14%)	1 (25%)	6 (13%)	0.46
Diabetes	4 (8%)	1 (25%)	3 (7%)	0.29
Fatty liver	1 (2%)	0 (25%)	1 (2%)	1
ALT (U/L)	26.8 ± 22.9	22.3 ± 8.2	15.0 ± 8.1	0.83
**Antirheumatic therapies before RTX**				
bDMARDs				
Abatacept	0 (0%)	0 (0%)	0 (0%)	
Adalimumab	22 (44%)	4 (100%)	18 (39%)	0.02
Duration (months)	19.2 ± 17.2	18.8 ± 11.9	19.3 ± 18.5	0.95
Cumulative dose (mg)	1551 ± 1139	1610 ± 915.9	1537.8 ± 1205.5	0.64
Etanercept	13 (26%)	0 (0%)	13 (28%)	0.56
Duration (months)	21.5 ± 19.61			
Cumulative dose (mg)	4221.2 ± 3606.5			
Golimumab	4 (8%)	0 (0%)	4 (9%)	0.99
Duration (months)	8.3 ± 8.1			
Cumulative dose (mg)	525 ± 427.3			
Tocilizumab	3 (6%)	0 (0%)	3 (7%)	0.99
Duration (months)	18.3 ± 21.5			
Cumulative dose (mg)	4200 ± 2458			
n of bDMARDs				0.24
0	14 (28%)	0 (0%)	14 (30%)	
1	30 (60%)	4 (100%)	26 (57%)	
2	6 (12%)	0 (0%)	6 (13%)	
3	0 (0%)	0 (0%)	0 (0%)	
sDMARDs				
Azathioprine	3 (6%)	0 (0%)	3 (7%)	0.99
Cyclosporine	16 (32%)	0 (0%)	16 (35%)	0.30
Leflunomide	21 (42%)	1 (25%)	20 (43%)	0.63
Methotrexate	48 (96%)	4 (100%)	44 (96%)	0.99
Sulfasalazine	44 (86%)	4 (100%)	40 (87%)	0.46
Glucocorticoid (oral)*	44 (88%)	4 (100%)	40 (87%)	0.99
Antirheumatic therapy at time of starting RTX				
Methotrexate	34 (68%)	3 (75%)	31 (67%)	0.98
Glucocorticoid (oral)*	40 (80%)	3 (75%)	37 (80%)	
Prednisolone equivalent dose (mg/day)	4.8 ± 3.3	5 ± 4	4.7 ± 3.3	0.89
High dose (>20 mg/day, ≥4 wk)	0 (0%)	0 (0%)	0 (0%)	
Moderate dose (10–20 mg/day, ≥4 wk)	9 (22%)	1 (33%)	8 (17%)	
Low dose (<10 mg/day, ≥4 wk)	31 (78%)	2 (67%)	29 (63%)	
Azathioprine	3 (6%)	0 (0%)	3 (6%)	0.99
Cyclosporine	5 (10%)	1 (25%)	4 (9%)	0.35
Leflunomide	13 (26%)	2 (50%)	11 (24%)	0.28
Sulfasalazine	35 (70%)	3 (75%)	32 (70%)	0.99

^#^Data values shown: mean ± SD or number (%).

^†^*p* < 0.05 (patients with vs. without HBV reactivation).

^*^Glucocorticoids (oral): cortisone, prednisolone, methylprednisolone, dexamethasone.

Abbreviations: HBV, hepatitis B virus; HCV, hepatitis C virus; HBsAg, HBV surface antigen; HBcAb, HBV core antibody; HBsAb, HBV surface antibody; NA, not available; RTX, rituximab; BMI, body mass index; sDMARDs, synthetic disease-modifying antirheumatic drugs; bDMARDs, biological DMARDs.

The average follow-up time was 3.9 years (SD, 2.5 years). RTX was administered for a mean of 5.7 cycles in combination with methotrexate (34 patients, 68%) or glucocorticoids (40 patients, 80%). The mean prednisolone equivalent dose was 4.8 mg/day, with all patients receiving a low to moderate dose of steroids. Sixty-three (72%) patients had been previously treated with 1 or more different biological drugs. Most patients were administered adalimumab (22 patients; 44%) or etanercept (13 patients; 26%).

### Demographics and baseline clinical features predisposing HBV reactivation in RA patients with HBsAg^−^/HBcAb^+^

**Figure 2:**
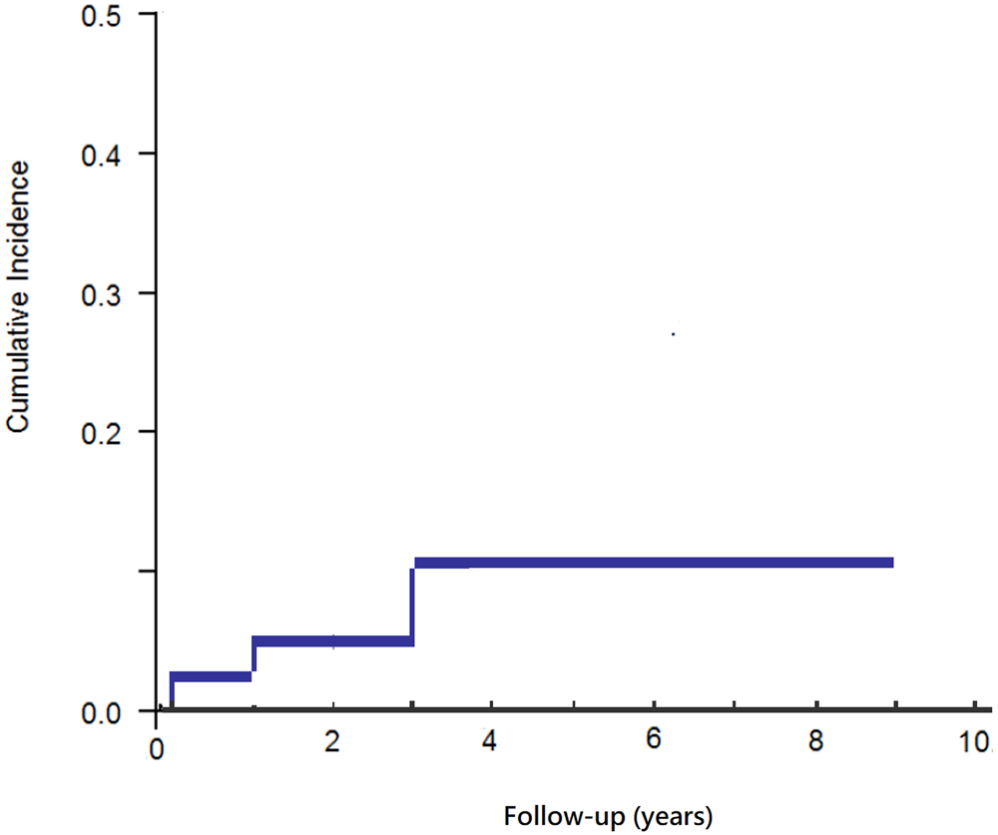
Cumulative risk of HBV reactivation following treatment.

With RTX administration, four patients (8%) experienced HBV reactivation after a mean duration of 3.5 years (SD, 2.5 years). Figure 2 shows the cumulative incidences of HBV reactivation following RTX treatment. In the HBV reactivation group, the mean age was 77.5 years. One patient had hepatitis C virus (HCV) coinfection (Table 1), with the HCV RNA level undetectable upon HBV reactivation (Table 2). Three patients (75%) had HBsAg seroreversion at the time of reactivation. HBV reactivation was significantly more common in baseline HBsAb^−^ patients than in HBsAb^+^ patients (30% vs 4%; *p* = 0.02), all of whom had been previously treated with adalimumab (100% vs 39%; *p* = 0.02). There were no significant differences between HBsAg^−^/HBcAb^+^ patients with and without HBV reactivation with respect to age, sex, cycle, previous use of sDMARDs, or concomitant use of methotrexate, prednisolone, or other sDMARDs during RTX therapy.

**Table 2:** Clinical features of patients (HBsAg^−^/ HBc Ab^+^) with RA who developed HBV reactivation during RTX therapy.

Characteristics	Case 1	Case 2	Case 3	Case 4
Age/sex	72/F	68/F	80/F	90/F
BMI (kg/m^2^)	16.9	18.1	32.9	17.6
Hypertension	without	without	without	with
Hyperlipidemia	without	without	without	with
Diabetes	without	with	without	without
RTX cycle	3	5	2	5
RTX dose (mg)	1000	1000	1000	1000
HBsAg seroreversion	+	−	+	+
HBsAb	−	+	−	−
Anti-HCV	−	+	−	−
HCV RNA (IU/mL) At baseline/At flare-up	−	ND/ND	−	−
HBV DNA (IU/mL)				
At baseline	NA	ND	ND	ND
At reactivation	1.38 × 10^8^	3.72 × 10^7^	1.78 × 10^7^	3.72 × 10^7^
ALT (U/L)				
At baseline	15	26	10	24
At reactivation	19	919	476	16
HBeAg at reactivation	NA	NA	−	NA
T.bil (mg/dl)	NA	0.5	16.8	NA
PT prolongation	NA	N	>50	NA
Ascites	N	N	Y	N
Hepatic encephalopathy	N	N	Y	N
Hepatitis flare up/reason	N	Y/HBV	Y/HBV	N
Time to reactivation (weeks)				
From first RTX	81	182	51	205
From Last RTX	23	23	26	85
Treatment for HBV reactivation	Entecavir	Entecavir	Entecavir	Entecavir
Outcome	alive & well	alive & well	Expire due to hepatitis	alive & well
Follow-up time (weeks)	98	251	55	333
Antirheumatic therapies before RTX				
SDMARDs	MTX/Pd/SSZ	MTX/Pd/LEF/SSZ	MTX/Pd/SSZ	MTX/Pd/SSZ
bDMARDs/(month)	ADA/9	ADA/30	ADA/32	ADA/12
Concomitant immune-suppressants				
MTX (mg/week)	Y/7.5	N	Y/12.5	Y/7.5
Prednisolone equivalent dose (mg/day)	5	0	10	5
SDMARDs	SSX	LEF/SSZ	N	CSA/LEF/SSZ

RA, rheumatoid arthritis; HBV, hepatitis B virus; HCV, hepatitis C virus; M, male; F, female; RTX, rituximab; HBsAg, HBV surface antigen; HBc Ab, HBV core antibody; HBsAb, HBV surface antibody; ALT, alanine aminotransferase; IU, international units; T.bil, total bilirubin; PT, prothrombin time; sDMARD, Pd, prednisolone; MTX, methotrexate; LEF, leflunomide; SSZ, sulfasalazine; bDMARDs, ADA, adalimumab; NA, not applicable; ND, target undetected; N, did not happen; Y, happened.

### Clinical features of patients with HBV reactivation

**Figure 3:**
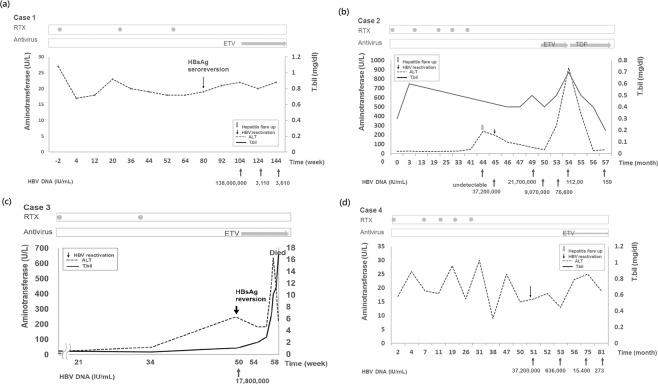
Time course of serum alanine aminotransferase (ALT) and total bilirubin (T.bil) levels in patients experiencing hepatitis B virus reactivation. RTX, rituximab; ETV, entecavir.

The clinical data from HBsAg^−^/HBcAb^+^ patients who experienced HBV reactivation are shown in Table 2 and Fig. 3. All these patients were female. The mean duration of RTX therapy was a 3.5 cycles. The mean age at HBV reactivation was 77.5 ± 9.7 years. HBV reactivation developed approximately 3.5 years after the initiation of RTX therapy. Three of the patients experienced HBV reactivation after discontinuation of the RTX regimen, with a mean duration of 39.3 ± 31 weeks from the last RTX infusion.

At the time of HBV reactivation, 3 of the patients underwent HBsAg seroreversion; the average HBV DNA level was 5.76 × 10^7^ (IU/mL). Two patients experienced HBV reactivation accompanied by hepatitis flare-up. All four patients received rescue therapy with entecavir, with three achieving viral suppression and recovering without complications. Patient 3 died due to complications from acute hepatic failure (Fig. 3).

## Discussion

The findings of our study indicate a moderate risk of HBV reactivation (4/50, 8%) in HBsAg^−^/HBcAb^+^ RA patients after treatment with RTX. All HBV reactivation occurred approximately 1–4 years after the first dose of RTX and approximately 0.5–1.5 years after the last RTX dose. A history of adalimumab administration or without HBsAb at baseline was associated with HBV reactivation.

HBV reactivation is a potentially life-threatening complication in patients receiving RTX therapy. The reported incidence of HBV reactivation in HBsAg^+^ patients with rheumatologic disease, particularly RA, is 12.3%^17^. However, the risk of HBV reactivation varies in HBsAg^−^/HBcAb^+^ patients receiving RTX for RA. The differences in findings between the few reports available may result from differences in follow-up time, sample size, monitoring policy, the definition of HBV reactivation and race. In an Italian multicenter study of patients receiving a median of 3 cycles of RTX (range, 1–8) over 34 months of follow-up (range, 0–80), the risk of HBV reactivation was 3% (1/33) in HBsAg^−^/HBcAb^+^ patients^16^. A cohort study from Taiwan with a follow-up of 3.4 ± 1.7 years from RTX initiation reported an HBV reactivation rate of approximately 9.1% (4/44)^15^, consistent with that observed in our study (8%; 4/50). It must be noted that HBV reactivation and hepatitis flare-up may occur months after stopping RTX; hence, the incidence data are greatly influenced by the length of follow-up^18^. Our study of 50 patients with a mean follow-up of 3.9 ± 2.5 years from the initiation of RTX may provide more accurate results.

In three of the HBV reactivation patients, the episodes happened around 6 months after the last dose of RTX, and in the other patient it was about 1.5 years. (Table 2) Several studies have explained the reasons for this variability^19,20^. Most episodes of HBV reactivation occur through a change in the balance between the host immunologic response to HBV and viral replication activity. The imbalance could occur through loss of immune control due to immunosuppressive therapy. HBV reactivation can occur after the cessation of immunosuppressive agents, presumably as the result of stimulation of HBV replication and restoration of host immune responses to infected hepatocytes.

Little is known about the risk, predictive factors, and clinical consequences of HBV reactivation in patients with HBsAg^−^/HBcAb^+^ treated with RTX for RA, and its management differs between clinical practice guidelines. HBsAb appears to be a protective factor against HBV reactivation. One recent meta-analysis demonstrated that HBsAb protects against reactivation in HBsAg^−^/HBcAb^+^ HBV patients receiving chemotherapy for hematological malignancies^21^. In 800 patients from 10 studies receiving only RTX-based chemotherapies, HBsAb^+^ patients had a lower rate of reactivation than did HBsAb^−^ patients (6.5% versus 23.7%), with an OR of 0.19 (95% CI, 0.11–0.31)^21^. Serum HBsAb titer may help determine when to administer antiviral treatment^22–24^. In one study of 108 patients with lymphoma treated with RTX, patients with high baseline HBsAb (≥100 mIU/mL) had a significantly lower risk of HBV reactivation (HR, 0.49; *p = *0.006) than those with low baseline anti-HBs (<100 mIU/mL)^22^. In our study, the presence of anti-HBsAb was associated with a lower risk of reactivation (4% versus 30%). Our results underscore the importance of monitoring HBsAb levels for risk stratification, but the cut-off level indicating high-risk serum HBsAb titers is unknown, requiring further study.

HBV reactivation in HBsAg^−^/HBcAb^+^ HBV patients after RTX treatment may be associated with a history of treatment with other bDMARDs before RTX^25^. In our study, 72% of the patients (36/50) had been treated previously with bDMARDs, and nearly all had received tumor necrosis factor (TNF)-alpha inhibitors (91%, 30/33) (Table 1). Adalimumab and etanercept were the most common bDMARDs administered before RTX. All the patients experiencing HBV reactivation had received adalimumab before RTX therapy. TNF-alpha is a proinflammatory cytokine that suppresses HBV replication and promotes HBV eradication by stimulating HBV-specific cytotoxic T-cell responses^26^. One systematic review of HBV reactivation in RA patients receiving TNF-alpha inhibitors reported that adalimumab carried a higher risk of HBV reactivation than etanercept (4.6% vs. 3.9%, respectively). The risk of tuberculosis is also higher in patients treated with adalimumab than in those treated with etanercept^5^. This difference may result from the weaker induction by etanercept of complement-dependent cytotoxicity on membrane-bound TNF-alpha-expressing cells^27^. The association between HBV reactivation and previous treatment with different TNF-alpha inhibitors is still unclear and requires further study. At present, we suggest the use of close monitoring or a prophylaxis strategy before starting RTX in HBsAg^−^/HBcAb^+^ patients with a history of TNF-alpha inhibitor treatment. In this study, the effect of previous treatment with golimumab or tocilizumab on HBV reactivation was not confirmed due to the small sample size, necessitating further study.

Our study shows no association between previous or concomitant use of sDMARDs and HBV reactivation. Conventional DMARDs are commonly used in the treatment of RA, and they seem to be relatively safe^28^. The long-term use of high doses of glucocorticoids is strongly associated with HBV reactivation in RA studies, resulting from its immune suppression activity and direct stimulation of HBV replication. Antiviral prophylaxis should be considered for those administered relatively high dosages of glucocorticoids (>20 mg/day for at least 4 weeks)^28^. A prospective study in Japan including 1330 HBsAg^−^ patients on immunosuppressive therapy for rheumatic disease indicated that prednisolone is a risk factor for HBV reactivation (RR, 2.2; 95% CI, 1.0–4.6)^24^. In our cohort, 80% of the patients (40/50) received steroids during RTX treatment. However, because most of our patients received a low dose (31/40 [78%]; <10 mg/day) and the sample size was small, a relationship between steroid treatment and HBV reactivation was not observed in our cohort.

HBV reactivation may occur ≥12 months after RTX use, probably due to extended immunosuppression and delayed recovery caused by RTX. In our study, HBV reactivation occurred approximately 3.5 years after the initiation of RTX therapy and a mean of 6 months after the last RTX dose. A previous study in Taiwan showed a mean time to HBV reactivation of approximately 2 years from the first dose of RTX^29^. The differences in these results may be related to the monitoring protocol. In our study, the patients had regular alanine aminotransferase (ALT)/ aspartate aminotransferase (AST) monitoring but were not tested frequently for HBsAg/HBV DNA. The diagnosis of HBV reactivation may have been delayed, resulting in more severe hepatitis. Of the 4 patients experiencing HBV reactivation in our study, 2 experienced hepatitis flare-up, one of them eventually dying from related complications (Table 2). Moreover, the time course of case 3 (Fig. 3) showed a long delay (16 weeks) between the first mild increase in ALT level (47 IU/ml at week 47) and the identification of HBsAg seroreversion. This delay, together with the very high viral load, determined the poor outcome of this patient. For those patients who received HBsAg/HBV DNA monitoring, two patients with HBV reactivation could be diagnosed by HBsAg seroreversion without an ALT increase. These results add to our current knowledge about the monitoring of HBV reactivation in patients with RA receiving RTX^11^.

There are several limitations to this study. First, some baseline data, such as HBeAg, anti-HBe status, HBsAb level and HBV DNA levels, were not available in this study. Because baseline HBV DNA was not available in every patient, the presence of occult HBV infection before RTX treatment cannot be completely ruled out. The prevalence of occult HBV infection varies greatly across the world and across patient populations^30^. In carefully conducted studies of blood donors, HBV DNA was detected in 0% to 4.6% of those who were HBsAg^−^/HBcAb^+^, with a median prevalence of 1%^30^. Missing data are common in a retrospective study, and the low prevalence of occult HBV infection may have had little impact on our results. Second, there is no strict follow-up protocol in our hospital, and the diagnosis of HBV reactivation may have been delayed, resulting in fulminant hepatitis. The risk of HBV reactivation may be underestimated. Our investigation includes the largest cohort of any study of this subject. Because of the large number of person-years of follow-up, our findings nonetheless provide important information regarding treatment decisions in this population. Third, some monitoring markers, such as HBsAb level, HBV genotype, and HBV core-related antigen, were not available in this study. These new markers need further study to confirm the role of HBV reactivation. Fourth, the long duration of this retrospective study covers many years in which strategies significantly evolved. With medical advances, medical care and monitoring policies may change. A long duration allowed us to observe the episodes of HBV reactivation and represent a real-world picture in an epidemic area.

## Conclusion

HBsAg^−^/HBcAb^+^ RA patients who receive RTX therapy are at moderate risk of HBV reactivation that may induce acute hepatitis flare-ups, even leading to death. Previous adalimumab use and HBsAb^−^ status may increase this risk. Regular monitoring of ALT/AST is insufficient; prophylaxis with antiviral treatment or monitoring of HBsAg and HBV-DNA should be performed to decrease the risk of HBV reactivation in RA patients treated with RTX.

## Methods

### Patients

The records of RA patients who had received RTX therapy between January 2000 and December 2017 at Dalin Tzu Chi General Hospital were reviewed to determine their eligibility for inclusion in this retrospective study. The inclusion criteria were as follows: age ≥18 years; availability of HBsAg and HBcAb status at diagnosis; and having received at least one cycle of RTX therapy. In accordance with international guidelines^1^, the standard treatment cycle of RTX was administered at an intravenous (IV) dose of 1000 mg on days 1 and 15 every 6 months or on the basis of the patients’ clinical responses. Patients were excluded if they were <18 years, received RTX for a reason other than RA treatment, had incomplete data, or received RTX for less than one cycle.

The study conformed to the ethical guidelines of the 1975 Declaration of Helsinki as reflected by a priori approval by the Ethics Committee of Dalin Tzu Chi General Hospital (approval number B10704017). Due to the retrospective design of the current study and anonymous analysis of the data, the requirement for informed consent was waived by the Ethics Committee of Dalin Tzu Chi General Hospital.

Of the initial 134 RA patients receiving RTX initially recruited, 87 remained after exclusion for missing data regarding HBsAg or HBcAb status (n = 36) or for receiving RTX for less than one cycle (n = 11) (Fig. 1). Of the remaining 87 patients, 7 were HBsAg^+^ and 80 were HBsAg^−^ (Fig. 1). Of the HBsAg^+^ patients, 2 were previously treated with antiviral agents (entecavir), one for spontaneous active chronic hepatitis B and the other for etanercept-related HBV reactivation. Three HBsAg^+^ patients who were not previously treated with antiviral agents experienced HBV reactivation during RTX therapy, an incidence of 60%. Of the HBsAg^−^ population, a total of 50 who were HBsAg^−^/HBcAb^+^ were enrolled in this study (Fig. 1).

### Follow-up of the study population

The medial chart was reviewed retrospectively. The baseline characteristics, including HBV serum markers (HBsAg, HBcAb, hepatitis B surface antibody [HBsAb], HBV DNA), anti-hepatitis C virus antibody (anti-HCV) and liver biochemical parameters (serum aspartate aminotransferase [AST]; alanine aminotransferase [ALT]), comorbidity, comedications, and the occurrence of HBV reactivation, were recorded. Patients were monitored for ALT and AST levels every 3 months; and HBsAg and HBV DNA tests were repeated whenever clinically indicated. The primary study endpoint was the HBV reactivation rate. HBV reactivation was defined as the detection of HBV DNA or the reappearance of HBsAg (seroreversion)^11^. The secondary endpoint was hepatitis flare-up, defined as an ALT increase of 3 times the baseline level and >100 U/L^11^. Detailed medical records were collected, including immunological profiles, RTX course, previous and concomitant DMARD therapy, and corticosteroid therapy.

### Statistical analysis

Categorical variables are presented as counts and percentages, and continuous variables are presented as the mean value and standard deviation (SD). The chi-squared test or Fisher’s exact test for categorical variables and Student’s t-test for continuous variables were used to compare the demographic and clinical characteristics between patients with and without HBV reactivation. All *p* values were 2-tailed, and p ≤ 0.05 was considered statistically significant. The data were statistically analyzed using SPSS 19.0 for Windows (SPSS Inc, Chicago, IL, USA).

## References

[1] Smolen JS (2017). EULAR recommendations for the management of rheumatoid arthritis with synthetic and biological disease-modifying antirheumatic drugs: 2016 update. Ann Rheum Dis.

[2] Holroyd CR (2019). The British Society for Rheumatology biologic DMARD safety guidelines in inflammatory arthritis. Rheumatology (Oxford).

[3] Tank ND, Karelia BN, Vegada BN (2017). Biological Response Modifiers in Rheumatoid Arthritis: Systematic Review and Meta-analysis of Safety. Journal of pharmacology & pharmacotherapeutics.

[4] Mori, S. & Fujiyama, S. Hepatitis B virus reactivation associated with antirheumatic therapy: risk and prophylaxis recommendations. **21**, 10274 (2015). 10.3748/wjg.v21.i36.10274 PMC4579875 26420955

[5] Lim CH (2017). The risk of tuberculosis disease in rheumatoid arthritis patients on biologics and targeted therapy: A 15-year real world experience in Taiwan. PLoS One.

[6] Reddy K. Rajender, Beavers Kimberly L., Hammond Sarah P., Lim Joseph K., Falck-Ytter Yngve T. (2015). American Gastroenterological Association Institute Guideline on the Prevention and Treatment of Hepatitis B Virus Reactivation During Immunosuppressive Drug Therapy. Gastroenterology.

[7] Seto Wai-Kay, Chan Thomas Sau-Yan, Hwang Yu-Yan, Mak Lung-Yi, Wong Danny Ka-Ho, Fung James, Liu Kevin Sze-Hang, Cheung Ka-Shing, Lai Ching-Lung, Kwong Yok-Lam, Yuen Man-Fung (2019). Monitoring and Treatment of Patients Undergoing Immunotherapy With Anti-CD20 Who are Exposed to HBV. Clinical Gastroenterology and Hepatology.

[8] Seto WK (2014). Hepatitis B reactivation in patients with previous hepatitis B virus exposure undergoing rituximab-containing chemotherapy for lymphoma: a prospective study. J Clin Oncol.

[9] Yeo W (2009). Hepatitis B virus reactivation in lymphoma patients with prior resolved hepatitis B undergoing anticancer therapy with or without rituximab. J Clin Oncol.

[10] Kusumoto S, Tanaka Y, Mizokami M, Ueda R (2009). Reactivation of hepatitis B virus following systemic chemotherapy for malignant lymphoma. Int J Hematol.

[11] Terrault NA (2018). Update on prevention, diagnosis, and treatment of chronic hepatitis B: AASLD 2018 hepatitis B guidance. Hepatology.

[12] Lampertico Pietro, Agarwal Kosh, Berg Thomas, Buti Maria, Janssen Harry L.A., Papatheodoridis George, Zoulim Fabien, Tacke Frank (2017). EASL 2017 Clinical Practice Guidelines on the management of hepatitis B virus infection. Journal of Hepatology.

[13] Barone M (2015). Safety of long-term biologic therapy in rheumatologic patients with a previously resolved hepatitis B viral infection. Hepatology.

[14] Mitroulis I, Hatzara C, Kandili A, Hadziyannis E, Vassilopoulos D (2013). Long-term safety of rituximab in patients with rheumatic diseases and chronic or resolved hepatitis B virus infection. Ann Rheum Dis.

[15] Tien YC, Yen HH, Chiu YM (2017). Incidence and clinical characteristics of hepatitis B virus reactivation in HBsAg-negative/HBcAb-positive patients receiving rituximab for rheumatoid arthritis. Clin Exp Rheumatol.

[16] Varisco V (2016). Low Risk of Hepatitis B Virus Reactivation in HBsAg-negative/Anti-HBc-positive Carriers Receiving Rituximab for Rheumatoid Arthritis: A Retrospective Multicenter Italian Study. J Rheumatol.

[17] Lee YH, Bae SC, Song GG (2013). Hepatitis B virus reactivation in HBsAg-positive patients with rheumatic diseases undergoing anti-tumor necrosis factor therapy or DMARDs. Int J Rheum Dis.

[18] Sebastiani M (2017). Italian consensus Guidelines for the management of hepatitis B virus infections in patients with rheumatoid arthritis. Joint Bone Spine.

[19] Hwang JP, Vierling JM, Zelenetz AD, Lackey SC, Loomba R (2012). Hepatitis B virus management to prevent reactivation after chemotherapy: a review. Supportive care in cancer.

[20] Aygen B (2018). Immunosuppressive therapy and the risk of hepatitis B reactivation: Consensus report. The Turkish Journal of Gastroenterology.

[21] Paul S (2017). Role of surface antibody in hepatitis B reactivation in patients with resolved infection and hematologic malignancy: A meta-analysis. Hepatology.

[22] Cho Y (2016). High titers of anti-HBs prevent rituximab-related viral reactivation in resolved hepatitis B patient with non-Hodgkin’s lymphoma. J Med Virol.

[23] Pei SN (2012). Analysis of hepatitis B surface antibody titers in B cell lymphoma patients after rituximab therapy. Ann Hematol.

[24] Fukuda W (2017). Incidence of hepatitis B virus reactivation in patients with resolved infection on immunosuppressive therapy for rheumatic disease: a multicentre, prospective, observational study in Japan. Ann Rheum Dis.

[25] Schwaneck, E. C. *et al*. Management of anti-HBc-positive patients with rheumatic diseases treated with disease-modifying antirheumatic drugs—a single-center analysis of 2054 patients. **37**, 2963–2970 (2018). 10.1007/s10067-018-4295-8 30238380

[26] Cantini F (2014). HBV Reactivation in Patients Treated with Antitumor Necrosis Factor-Alpha (TNF-alpha) Agents for Rheumatic and Dermatologic Conditions: A Systematic Review and Meta-Analysis. International journal of rheumatology.

[27] Baddley JW (2018). ESCMID Study Group for Infections in Compromised Hosts (ESGICH) Consensus Document on the safety of targeted and biological therapies: an infectious diseases perspective (Soluble immune effector molecules [I]: anti-tumor necrosis factor-alpha agents). Clin Microbiol Infect.

[28] Choi J, Lim YS (2017). Characteristics, Prevention, and Management of Hepatitis B Virus (HBV) Reactivation in HBV-Infected Patients Who Require Immunosuppressive Therapy. The Journal of infectious diseases.

[29] Tien YC (2018). Changes in hepatitis B virus surface antibody titer and risk of hepatitis B reactivation in HBsAg-negative/HBcAb-positive patients undergoing biologic therapy for rheumatic diseases: a prospective cohort study. Arthritis Res Ther.

[30] Raimondo, G. *et al*. Update of the statements on biology and clinical impact of occult hepatitis b virus infection. *Journal of hepatology* (2019). 10.1016/j.jhep.2019.03.034 31004683

